# Spatial Analysis and Comparison of the Economic Burden of Common Diseases: An Investigation of 5.7 Million Rural Elderly Inpatients in Southeast China, 2010–2016

**DOI:** 10.3389/fpubh.2021.774342

**Published:** 2021-11-17

**Authors:** Xuwei Tang, Xiaoxu Xie, Zhixiang Rao, Zhenquan Zheng, Chanchan Hu, Shanshan Li, Zhijian Hu

**Affiliations:** ^1^Department of Epidemiology and Health Statistics, School of Public Health, Fujian Medical University, Fuzhou, China; ^2^Institute of Health Research, School of Public Health, Fujian Medical University, Fuzhou, China

**Keywords:** rural China, older adults, catastrophic health expenses, geographically weighted regression, age-related diseases

## Abstract

**Background:** As China embraced an aging society, the burden of age-related diseases had increased dramatically. Knowledge about spatial distribution characteristics of disease burden and the influencing factors of medical expenditure is of great significance to the formulation of health policies. However, related research in rural China is still insufficient.

**Methods:** A total of 5,744,717 records of hospitalized rural elderly in southeast China were collected from 2010 to 2016. We described the temporal trends of hospitalization medical expenditure and the prevalence of catastrophic health expenses (CHE) in the rural elderly by common diseases. Then, geographical information tools were used for visualization of geographic distribution patterns of CHE, the ordinary least squares methods (OLS) and geographically weighted regression (GWR) were employed to examine the influencing factors of medical expenditure.

**Results:** The number of CHE hospitalizations and the total number of hospitalizations for the rural elderly people increased by 2.1 times and 2.2 times, respectively, from 2010 to 2016. Counties with a high prevalence of CHE were clustered in the eastern coastal area (Moran's *I* = 0.620, *P* < 0.001, General *G* < 0.001, *P* < 0.001). Unspecified transport accidents, cardiovascular disease, and essential hypertension were the top causes of CHE in the rural elderly. Adequate hospital beds (*P* < 0.05) and reasonable utilization and distribution of town-level (*P* < 0.001) and county-level hospitals (*P* < 0.001) may help reduce medical expenditures.

**Conclusions:** In the context of an aging society, the disease burden for the elderly in rural areas should arouse more attention. These findings highlight the importance of age-related disease prevention and the rational allocation of medical resources in rural areas.

## Introduction

China is the country with the largest population and the largest number of older adults in the world, who began to embrace an aging society in 1999, is one of the first developing countries to enter an aging society. The population of China aged 65 years and over is about 150 million, accounting for 11.9% in 2018. Compared with the national census in 2010, the population aged 65 years and over increased by 33.7% (1). As the population is growing and aging, the age-related disease burden is rising rapidly. For instance, in the past two decades, the causes of disease burden in China have changed dramatically. Stroke and ischemic heart disease were the leading causes of death and disability-adjusted life-years (DALYs). Stroke, ischemic heart disease, lung cancer, chronic obstructive pulmonary disease, and liver cancer were the five leading causes of (years of life lost) YLLs. Musculoskeletal disorders, mental health disorders, and sense organ diseases were the three leading causes of years lived with disability (YLDs) (2). Besides, past research indicates that the aging problem was worse, and the burden of disease was higher in rural China (3, 4). A recent study found that residents in rural areas suffered a greater financial burden from health expenditures, with the percentage 2.4 times higher than that of urban residents in 2013 (5). Therefore, to cope with the challenge of aging to the health care system in rural areas, it is necessary to formulate disease-focused health policies. Taking corresponding preventive measures for common diseases of the elderly is an excellent way to reduce the burden of diseases. However, in China, less research has been done to compare common disease burden for the rural elderly.

Catastrophic health expenditures (CHE) mean that medical expenditures account for disposable income unreasonably, which is an important indicator to measure the economic burden of disease (6, 7). Previous studies have reported that rural areas have a higher incidence of CHE than urban areas, although China had made progress in reducing CHE (8), and related research also found that the elderly are a high-risk group of CHE in China (9, 10). Therefore, research on pointing out the causes of CHE in the elderly in rural areas is of great significance for taking measures to reduce CHE incidence. However, there exists a knowledge gap on CHE in common diseases for the rural elderly. Besides, to provide a reference for the formulation of health policies or disease prevention measures in different regions, spatial analysis has been widely used in epidemiology and health economics. For example, previous research applied spatial analysis to the description of geographic distribution characteristics of health resources such as hospitals, hospital beds, and medical experts, and diseases such as COVID-19 (11–13). However, there are few spatial analysis studies on the medical economic burden for the elderly in rural China, which is worthy of further exploration.

Accordingly, to provide references for health policymaking, our research investigated the rural elderly inpatients in southeast China from 2010 to 2016 and described the number of hospitalizations and the medical expenditures of common diseases of the elderly. In addition, the geographic spatial distribution characteristics of the prevalence of CHE in the rural elderly and the influencing factors of medical expenses were explored through spatial analysis.

## Methods

### Data Sources

The data of inpatients was extracted from the New Rural Cooperative Medical Scheme (NRCMS) in the Fujian province, a representative coastal province with about a population of 39 million and a land area of 121,400 km^2^ in southeast China. The NRCMS was guided, organized, and supported by the government, and rural residents participated voluntarily. The previous study indicated that China had accomplished nearly universal insurance coverage, the enrolment rate of NRCMS among rural residents had reached 96.6% in 2010 (14). Taking into account the privacy issues of inpatients, the data manager removed the names and phone numbers and Chinese ID numbers of inpatients, and the addresses of inpatients were reserved at the county level. The subjects of this study were rural residents, 60 years or older, and who participated in the NRCMS. Finally, 5,744,717 inpatient records were enrolled in the study. Variables included gender, age, residential county, low-income or not (low-income patients were defined as patients whose household per capita income was below the local minimum living standard), admitted hospital level (town, county, municipal, and provincial), year of admission, length of stays, discharge diagnoses according to the International Classification of Diseases Tenth Revision (ICD-10 code), surgery or not, year of admission, total medical fee, and medical insurance reimbursement amount. The demography, economics, traffic conditions, and health resources data of the counties in the study regions were extracted from the Statistics Yearbook (15). Variables include rural population (RP), total population, per capita income (PCI), per capita consumer expenditure (PCCE), per capita GDP (PGDP), total road length, land area, number of hospitals beds, and number of health technicians.

### Variables of Interest

The out-of-pocket (OOP) expenditure is defined as the differences between total medical fee and medical insurance reimbursement amount, and in spatial analysis, defined as the mean value of inpatients in the county. CHE is defined as OOP medical expenditure equal to or exceeded 40% of a household's capacity to pay (16). The capacity of inpatients to pay was estimated by the rural PCI and rural PCCE of the county where he lived when he was admitted. Road density (RD) is defined as the total length of roads in the county (km) divided by the land area of the county (km^2^). Hospital beds per thousand population (HB) is defined as the number of hospital beds in the county divided by whose total population. Health technicians per thousand population (HT) is defined as the number of health technicians in the county divided by whose total population. The proportion of town-level hospitals inpatients (PTH) is defined as the proportion of patients hospitalized in town-level hospitals to all inpatients in the county. The proportion of county-level hospitals inpatients (PCH) is defined as the proportion of patients hospitalized in county-level hospitals to all inpatients in the county. The measurements of our dependent and explanatory variables are presented in Supplementary Table 1.

### Data Analysis

Numbers (percentages) of inpatients were calculated for categorical variables. The numbers of inpatients with CHE, the prevalence of CHE, and the means of OOP expenditures by the top 50 discharged diagnose in each year were calculated and then visualized by heatmap and treemap. Global Moran's I was used to assess the spatial autocorrelation of the prevalence of CHE (17). Evidence of local clusters characteristics was evaluated using Anselin local Moran's I statistic (17). Clustering for the prevalence of CHE density was assessed by the Getis–Ord General G statistic (18). Hot spot analysis for the prevalence of CHE was piloted using Getis–Ord Gi^*^ (19, 20). Ordinary least square (OLS) regression was employed to examine the relationships between variables and OOP expenditure and perform the multiple co-linear diagnoses. To explore the spatial variability between the OOP expenditure and variables, geographically weighted regression (GWR) was adopted (21, 22). The data analysis was done using SPSS and Excel. Spatial analysis was done using ArcMap. Statistical significance was based on two-sided tests and was set to 5%.

## Results

### Demographic Characteristics

**Figure 1:**
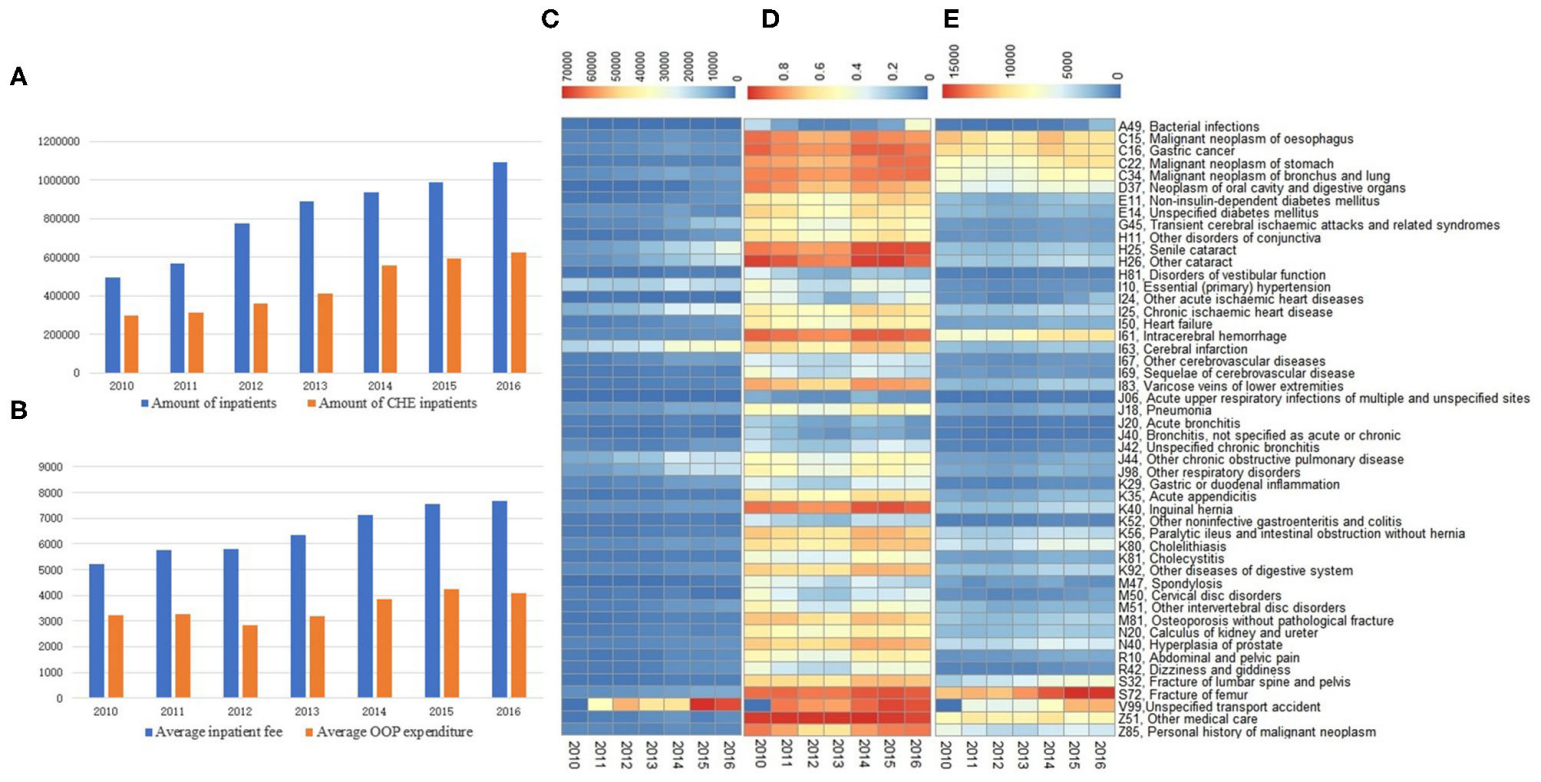
Medical expenditures and prevalence of catastrophic health expenses (CHE) of rural elderly inpatients, from 2010 to 2016. **(A)** Amounts of inpatients and inpatients with CHE. **(B)** Average inpatient fee and out-of-pocket (OOP) expenditure of inpatients, unit Yuan. **(C)** Amounts of inpatients with CHE in the top 50 discharge diagnoses. **(D)** The prevalence of CHE in the top 50 discharge diagnoses. **(E)** Average OOP expenditure in the top 50 discharge diagnoses, unit Yuan.

Table 1 shows the demographic characteristics of rural elderly inpatients in southeast China from 2010 to 2016. In total, there were 5,744,717 hospitalizations; 52.6% of inpatients aged older than 70 years, 49.6% were admitted for more than 1 week, and 14.6% of the patients underwent surgery. The number of inpatients in the town, county, municipal, and provincial hospitals accounted for 38.8, 36.1, 18.4, and 6.7% of the total, respectively. Besides, ~3 million (54.9% of all inpatients) may suffer CHE. Among all inpatients with CHE, there were 51% aged over 70 years, town, county, city, and provincial hospitals accounted for 36.8, 63.2, 76.7, and 23.3% respectively. Figures 1A,B illustrates that the number of inpatients and CHE inpatients had increased by 2.2 and 2.1 times, hospitalization expenses had increased by ~1.4 times, and OOP medical expenses had increased by ~1.3 times in the rural elderly from 2010 to 2016.

**Table 1:** Demographic characteristics of rural elderly inpatients, from 2010 to 2016, *n* (%).

**Variable**		**Inpatients (*n* = 5,744,717)**	**CHE inpatients (*n* = 3,151,087)**
Age (years)	60–63	1,244,209 (21.7)	712,556 (22.6)
	64–69	1,478,095 (25.7)	828,964 (26.3)
	70–76	1,513,536 (26.3)	807,918 (25.6)
	≥77	1508,877 (26.3)	801,649 (25.4)
Gender	Female	2,824,879 (49.2)	1,494,135 (47.4)
	Male	2,915,970 (50.8)	1,655,260 (52.5)
Low-income households	No	5,605,879 (97.6)	3,084,230 (97.9)
	Yes	138,838 (2.4)	66,857 (2.1)
Hospital level	Town	2,230,231 (38.8)	185,808 (5.9)
	County	2,074,820 (36.1)	1563540 (49.6)
	Municipal	1,057,237 (18.4)	1,024,168 (32.5)
	Provincial	382,393 (6.7)	377,537 (12.0)
Length of stays (days)	0–6	2,897,462 (50.4)	1,160,531 (36.8)
	≥ 7	2,847,175 (49.6)	1,990,515 (63.2)
Surgery	No	4,910,987 (85.5)	2,417,970 (76.7)
	Yes	833,730 (14.5)	733,117 (23.3)

### CHE in Top 50 Discharge Diagnoses

**Figure 2:**
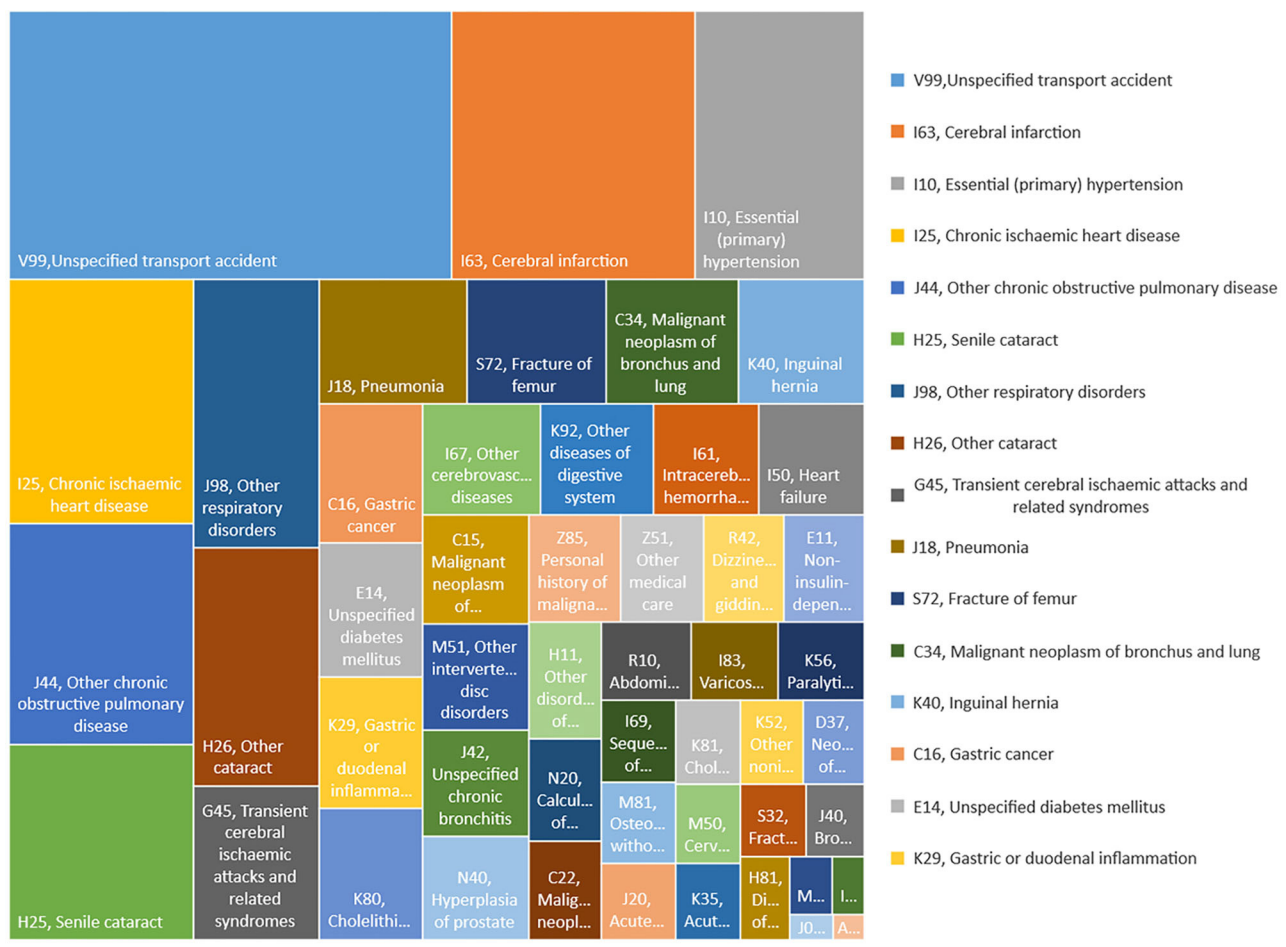
The proportion of rural elderly inpatients with CHE by discharge diagnoses from 2010 to 2016.

The number and prevalence of CHE and the average OOP medical expenditure caused by the top 50 discharge diagnoses from 2010 to 2016 are shown in Figures 1C–E, in which the differences in the number of inpatients, prevalence rates of CHE, average OOP expenditure between years, and discharge diagnoses are visualized by colors. Exactly, during the study period, unspecified transport accidents, senile and other cataracts, cerebral infarction, and chronic ischemic heart disease were the discharge diagnoses with the most significant increase in the actual number of inpatients with CHE, among the top 50 discharge diagnoses (Figure 1C). Meanwhile, other medical care, other cataracts, fracture of the femur, intracerebral hemorrhage, senile cataract, and unspecified transport accidents accompanied the highest prevalence of CHE among the top 50 discharge diagnoses (Figure 1D). Besides, fracture of the femur, other medical care, intracerebral hemorrhage, unspecified transport accident, and cancers were the discharge diagnosis with the heaviest OOP expenditure in the top 50 (Figure 1E). In total, unspecified transport accidents, cerebral infarction, essential (primary) hypertension, chronic ischemic heart disease, and others such as chronic obstructive pulmonary disease were the top discharge diagnoses for CHE (Figure 2).

### Geographic Variation of CHE

**Figure 3:**
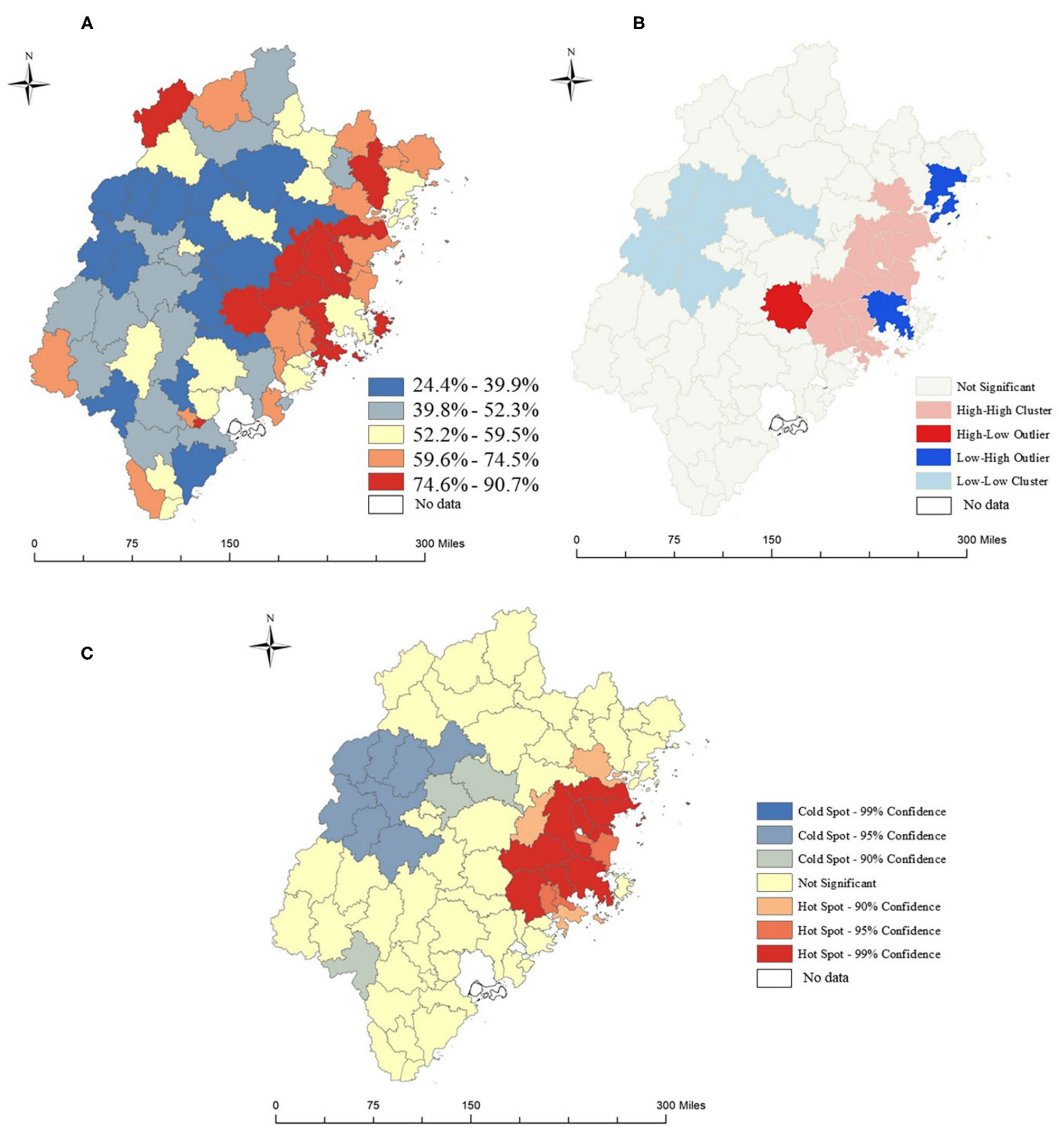
Spatial analysis of CHE prevalence among rural elderly inpatients in southeast China, from 2010 to 2016. **(A)** The prevalence of rural elderly inpatients with CHE. **(B)** Anselin local Moran's I analysis of the prevalence of rural elderly inpatients with CHE. **(C)** Hot spots analysis of the prevalence of rural elderly inpatients with CHE.

Figure 3A depicts the prevalence of CHE in southeast China during the study period at the county level. The results of the spatial analysis of the prevalence of CHE are shown in Table 2, in which the result of the spatial analysis of Global Moran's I was 0.620, the *Z* = 8.329, and the *P* < 0.001, indicating significant spatial autocorrelation for the prevalence of CHE in the study area. Furthermore, the observed value of General G < 0.001, *Z* = 4.598, *P* < 0.001, indicates that there were hot or cold spot areas of CHE prevalence within the scope of the study (Table 2). In addition, Figure 3B displays the local clusters of the prevalence of CHE by using the local Moran's I, which showed that the high–high cluster regions were located in east coast county, and on the contrary, the low–low cluster regions located in western Inland county. Likewise, Getis–Ord Gi^*^ analysis results show that hot spot regions (99% confidence) of the prevalence of CHE were gathered in the east coast, and cold spot regions (99% confidence) gathered in the east (Figure 3C).

**Table 2:** Summary of Global Moran's I and Getis–Ord General G results.

**Global Moran's I**		**Getis–Ord General G**	
**Parameter**	**Estimated value**	**Parameter**	**Estimated value**
Expected index	−0.002	General G observed value	<0.001
Moran's I	0.620	General G expected value	<0.001
Variance	0.006	Variance	<0.001
*Z*	8.329	*Z*	4.598
*P*	<0.001	*P*	<0.001

### Factors Related to OOP Expenditure

Table 3 and Supplementary Figure 1 show the results of the OLS model in 2011, 2013, and 2015. The R-squared of the model were 0.87, 0.81, and 0.81, and the standard deviation of most counties is controlled within 1.5 times, except two counties in 2013, which represented a good model fit to OOP expenditure of the rural elderly in each county of the study area. Results reveal that the lower OOP expenditure of each county was related to more HB (coefficient = −281.38, *P* = 0.024, in 2011; coefficient = −378.25, *P* = 0.004, in 2013; coefficient = −339.82, *P* = 0.013, in 2015), the county with more HT suggest higher OOP expenditure (coefficient = 324.23, *P* = 0.001, in 2011; coefficient = 263.02, *P* = 0.023, in 2013), and the county with a higher PTH prompt lower OOP expenditure (coefficient = −53.54, *P* < 0.001, in 2011; coefficient = −74.11, *P* < 0.001, in 2013; coefficient = −84.69, *P* < 0.001, in 2015). County with a higher PCH prompt lower OOP expenditure (coefficient = −27.85, *P* < 0.001, in 2011; coefficient = −40.72, *P* < 0.001, in 2013; coefficient = −44.04, *P* < 0.001, in 2015).

**Table 3:** Summary of ordinary least squares methods and geographically weighted regression results.

**Year**	**Variable**	**OLS**				**GWR**	
		**Coefficient^a^**	**P^b^**	**VIF^c^**	**R-squared^d^**	**Coefficient, mean (min, max)**	**R-squared^d^**
2011	Intercept	3,912.96	0.000^**^	–	0.87	4,275.198 (4,211.795, 4,291.947)	0.84
	RP	13.84	0.009^*^	2.32		7.708 (7.608, 7.818)	
	PCI	0.18	0.019^*^	4.15		0.220 (0.216, 0.223)	
	PCCE	−0.01	0.93	4.64		–	
	PGDP	−57.67	0.456	3.68		–	
	RD	578.68	0.008^*^	2.28		418.646 (412.420, 424.743)	
	HB	−281.38	0.024^*^	7.77		–	
	HT	324.23	0.001^*^	8.95		–	
	PTH	−53.54	0.000^**^	2.1		−58.821 (−58.952, −58.723)	
	PCH	−27.85	0.000^**^	2.22		−33.545 (−33.658, −33.420)	
2013	Intercept	6,566.93	0.000^**^	–	0.81	8,770.820 (5,924.285, 10,610.617)	0.89
	RP	11.63	0.081	1.77		–	
	PCI	0.08	0.277	3.24		–	
	PCCE	0.08	0.384	4.89		–	
	PGDP	−47.01	0.509	3.03		–	
	RD	−182.07	0.458	1.85		–	
	HB	−378.25	0.004^*^	7.43		−162.626 (−420.421, 60.037)	
	HT	263.02	0.023^*^	8.22		–	
	PTH	−74.11	0.000^**^	2.64		−80.781 (−90.687,−56.239)	
	PCH	−40.72	0.000^**^	2.2		−49.103 (−57.715, −30.667)	
2015	Intercept	6,585.25	0.000^**^	–	0.81	6,781.457 (6,436.422, 7,129.868)	0.80
	RP	4.28	0.628	3.28		–	
	PCI	−0.02	0.897	6.15		–	
	PCCE	0.33	0.018^*^	6.46		0.276 (0.264, 0.286)	
	PGDP	−79.21	0.316	3.01		–	
	RD	−34.69	0.83	2.66		–	
	HB	−339.82	0.013^*^	6.33		−215.472 (−242.811, −185.599)	
	HT	160.65	0.952	7.11		–	
	PTH	−84.69	0.000^**^	1.72		−86.238 (−87.971, −84.131)	
	PCH	−44.04	0.000^**^	1.74		−44.835 (−45.220, −44.392)	

**Coefficient^a^:** *: represents the strength and type of relationship between each explanatory variable and the dependent variable*.

**Probability^b^:** *: the asterisk*

(*)or (**) *indicates a coefficient is statistically significant (p < 0.05) or (p < 0.001)*.

**Variance inflation factor^c^:** *(VIF): large VIF values (>7.5) indicate redundancy among explanatory variables*.

**The R-squared^d^:** *: the fraction of the variance in the data that is explained by the model*.

*OLS, ordinary least squares methods; GWR, geographically weighted regression; RP, rural population; PCI, per capita income; PCCE, per capita consumer expenditure; PGDP, per capita GDP; RD, road density; HB, hospital beds per thousand population; HT, health technicians per thousand population; PTH, proportion of town-level hospitals inpatients; PCH, proportion of county-level hospitals inpatients*.

To eliminate the influence of collinearity, the variables with variance inflation factors (VIF) less than 7.5 and whose coefficients were statistically significant (*P* < 0.05) were included in the GWR model. Results for the GWR model can be seen in Table 3. To analyze the influence of each variable on OOP expenditures in each county, the coefficient distribution map for the influencing factors was shown in Supplementary Figure 2. Regression coefficient of PTH exhibited a characteristic of being “high in the southwest, low in the northeast” (coefficient ranged from −58.952 to −58.723 in 2011; from −90.687 to −56.239 in 2013; and from −87.971 to −84.131 in 2015) suggesting that the OOP medical expenditure of counties in the northeast are more sensitive to changes in PTH. Similarly, the coefficient of HB increased from southwest to northeast (coefficient ranged from −420.421 to 60.037 in 2013 and from −242.811 to −185.599 in 2015) suggesting that increasing the HB of counties in the northwest region may have higher effectiveness for reducing OOP medical expenditure. Besides, there was a rather unexpected outcome that the region with the lowest regression coefficient of PCH gradually shifted from the southwest to the northeast during the study period. Specifically, counties in the southwest region had the lowest PCH regression coefficient in 2011 (range from −33.658 to −33.420), counties in the northeast of the central region had the lowest in 2013 (range from −57.715 to −30.667), and in 2015 the northeast region had the lowest (range from −45.220 to −44.392). These outcomes suggest that in the future, increasing the PCH of counties in the northeastern region may have good benefits in reducing the OOP medical expenditure.

## Discussion

Findings from the research indicate that the hospitalization expenses of the rural elderly in southeast China showed a rapid growth trend during the study period. At the same time, the number of hospitalizations for the rural elderly and the number of hospitalizations for the rural elderly who suffered CHE also increased significantly. Counties with a high prevalence of CHE are clustered in the eastern part of the study area. Besides, we found that unspecified transport accidents, cerebral infarction, essential (primary) hypertension, chronic ischemic heart disease were the leading cause of CHE. Other medical care, other cataracts, and fracture of the femur were the discharge diagnoses with the highest prevalence of CHE for rural elderly inpatients. In addition, our research suggests that the OOP medical expenditures decreased with increasing HB, PTH, and PCH, which was more dramatic in the northeast region.

The finding of the study shows that the number of inpatients for rural elderly had increased by 2.2 times from 2010 to 2016. It is a particularly shocking result which means that the demand of the rural elderly for medical services had significantly increased. The observed increase in the number of inpatients could be attributed to the rise in the proportion of the elderly population in rural areas and the advance of the primary health system in China. The aging problem will pose a huge challenge to China's health care system. Prior studies have noted that with aging in China in the twenty first Century, the prevalence and incidence of age-related diseases have increased sharply. At the same time, a large number of young rural laborers have migrated to urban areas, resulting in an increase in the proportion of the rural elderly population and further aggravating the burden of the current rural health care system (3). Another possible explanation for increasing inpatients is that China's long-term health care reforms have achieved certain results. China launched 125 billion US dollars in 2009 to establish a national essential medicines system covering 90% of China's population by 2020, enabling more rural elderly to receive medical services and thus increasing the number of hospitalizations (14, 23). However, the current health care system faces the problems of waste of resources, low efficiency, poor quality, and the scarcity and unequal distribution of qualified labor (24). Therefore, the rational use and distribution of medical or health resources and the improvement of health care service quality are the primary goals of Chinese policymakers.

Another important finding is that the number of elderly who were hospitalized in the study area who suffered from CHE increased by 2.1 times during the study period. Counties with a high CHE prevalence were clustered in the eastern coastal region. These spatial distribution characteristics of CHE may partly be explained by the unequal distribution of medical and health resources. High-quality medical institutions, such as provincial and municipal hospitals, are mainly located in relatively developed coastal areas, which has caused residents in the eastern region to bear higher medical costs. Another possible explanation for this was that the local health system does not make full use of primary hospitals such as town-level hospitals and county-level hospitals, leading to a large number of patients with mild symptoms who choose high-level hospitals, which exacerbates the occurrence of CHE in the eastern regions. It is worth noting that among the 50 most diagnosed diseases, unspecified transport accidents, cerebral infarction, essential (primary) hypertension, and chronic ischemic heart disease were the leading causes of CHE.

Although traffic accidents are a major global public safety and health issue, what shocked us was that transport accidents, as a kind of injury accident, caused the most CHE for the elderly in rural areas, surpassing all diseases. Related research had found that China is in the most rapid development stage of road transportation. The number of vehicles, the number of motor vehicle drivers and car drivers, and the density of roads are increasing rapidly, leading to a significant increase in road accidents and deaths (25). Another study had shown that elderly people and rural area were the main factors associated with prehospital cardiopulmonary arrest due to a traffic crash (26). Therefore, road safety risk prevention and control, road safety legislation, road safety supervision, and road safety publicity and education should be taken seriously, especially in rural areas. Besides, as mentioned above, with the rise in aging population in China, the prevalence and incidence of these diseases had significantly increased.

Research results also suggest that circulatory system diseases, such as cerebral infarction, essential hypertension, and chronic ischemic heart disease are also the leading causes of CHE for rural residents. This finding could have been generated by the increased cardiovascular disease (CVD) treatment and informal caregiving costs. A similar situation has been found in the United States. In the United States, people over the age of 65 years spend the most on CVD, and the total cost of CVD increased to $616 billion in 2015 and is expected to increase to $1.2 trillion by 2035 (27).

We found that the diagnosis with the highest prevalence of CHE was other medical care, other cataracts, and fracture of the femur. According to ICD-10, other medical terms include radiotherapy session, chemotherapy session for neoplasm, maintenance chemotherapy, blood transfusion, preparatory care for subsequent treatment, palliative care, desensitization to allergens, preparatory care for subsequent treatment, and palliative care. Those treatments are mainly applied to advanced tumors or other diseases with very high mortality, low incidence, and require high costs. Affected by the age factor, the elderly are more prone to fracture injuries. Fractures need to be treated for a long time, which brings a huge burden to individuals and the society. Recent studies in China suggest that the total number of hip fractures aged 55 years and older increased by about 4 times. The total hospitalization cost per patient increased by about 1.59 times between 2012 and 2016 (28). Research on the medical expenditure of cataracts was less reported. A cataract is an age-related disease and requires surgical treatment, and the medical insurance compensation rate being low, may cause a higher economic burden on the elderly.

In general, to reduce the prevalence of CHE among the elderly in rural areas, the health care system should be further improved, such as making full use of primary health institutions to improve the efficiency of medical services, especially in the eastern regions where CHE is more serious. Second, take measures for the top diseases that cause CHE and the diseases with a high prevalence of CHE noted by the research. For example, by improving the living environment and focusing on health education, guiding the rural elderly to learn a healthy lifestyle reduces the prevalence of CHE-related diseases. On the other hand, the public medical insurance management department can flexibly adjust the hospital reimbursement rate based on economic conditions. For example, it is necessary to increase the compensation rate of medical insurance for areas or diseases with high prevalence of CHE.

The research results also manifest that the average OOP medical expenditures for the rural elderly inpatients in each county were associated with HB, PTH, and PCH. And there was a spatial difference in this relationship, which suggests that a reasonable allocation of medical resources in a specific area may be more efficient in reducing medical costs.

Hospital beds per thousand population is an indicator that can directly reflect the abundance of medical resources. The results demonstrated that increasing HB could help reduce local OOP medical expenditure for inpatients. This phenomenon was even more pronounced in the northeast. This phenomenon can be explained as follows. In China, there is an unequal distribution of medical resources. Health resources are mainly concentrated in economically developed areas. Affected by the level of consumption, medical expenses in financially developed areas are usually higher. Those have been reported in previous studies (24, 29). At the same time, residents in the northeast areas with insufficient medical resources have to visit other counties or cities with abundant medical resources to seek medical services that meet their needs and bear the high medical expenses that do not match their income.

The proportion of town- and county-level hospitals inpatients reflects the willingness of residents to seek medical services from local primary medical institutions, which may be affected by the quantity, quality, and efficiency of local primary medical institutions. The results also suggest that increasing PTH and PCT could help reduce local OOP medical expenditure, especially in the northwest region. Although compared with provincial and municipal medical institutions, the service quality of primary medical institutions is slightly inferior, and primary hospitals used to be characterized by high efficiency and low prices, which can meet the needs of health care for the most elderly. Therefore, prioritizing the development of primary medical institutions has shown superior benefits in reducing medical expenses.

## Limitations

There are some limitations to our study. First, we used rural per capita income and rural per capita consumer expenditure of each county to estimate the individual disposable income, which may bias the estimation of the prevalence of CHE. Because medical treatment may be affected by personal disposable income, wealthy people may be more willing to receive treatment. Second, under the influence of policies, some counties did not participate in NRCMS or withdrew halfway through, resulting in missing data, which may reduce the model fitting of OLS and GWR. Third, only the main diagnosis of the patient was recorded in the database, but in fact, many patients often suffer from multiple diseases at the same time. Therefore, the number of hospitalizations and the prevalence of CHE may be underestimated.

## Conclusions

In the context of aging, the number of hospitalizations, the number of hospitalizations with CHE, and the economic burden of the elderly in rural areas had increased. To reduce the economic burden of diseases for the elderly in rural areas, policymakers should pay attention to the prevention of traffic accidents, cardiovascular diseases, tumors, fractures, and cataracts, reasonably adjust the proportion of medical insurance compensation rate for the above diseases, and equitably allocate medical resources, especially in rural areas.

## Data Availability Statement

The datasets used in this study are available from the corresponding author on reasonable request.

## Author Contributions

ZH and XX conceptualized and led the study. XT and XX designed the study. Data collection was done by XT and ZZ. XT and ZR contributed to data analysis. The first draft of the article was written by XT. ZH, XX, CH, and SL reviewed the manuscript and provided critical inputs. All authors contributed to the article and approved the submitted version.

## Funding

This work was supported by the National Key R&D Program of China (Grant/Award Number: 2017YFC0907100).

## Conflict of Interest

The authors declare that the research was conducted in the absence of any commercial or financial relationships that could be construed as a potential conflict of interest.

## Publisher's Note

All claims expressed in this article are solely those of the authors and do not necessarily represent those of their affiliated organizations, or those of the publisher, the editors and the reviewers. Any product that may be evaluated in this article, or claim that may be made by its manufacturer, is not guaranteed or endorsed by the publisher.

## Supplementary Material

Supplementary Figure 1 Spatial distribution of the standard deviation of the OLS model.

Supplementary Figure 2 Spatial distribution of the coefficient of explanatory variables.

Supplementary Table 1 Definition and measurements of variables.
