# Supplementary figures and images for: Spatial Analysis and Comparison of the Economic Burden of Common Diseases: An Investigation of 5.7 Million Rural Elderly Inpatients in Southeast China, 2010–2016
Source: Front Public Health. 2021 Nov 17;9:774342. doi: 10.3389/fpubh.2021.774342 (PMC8635627; doi:10.3389/fpubh.2021.774342)

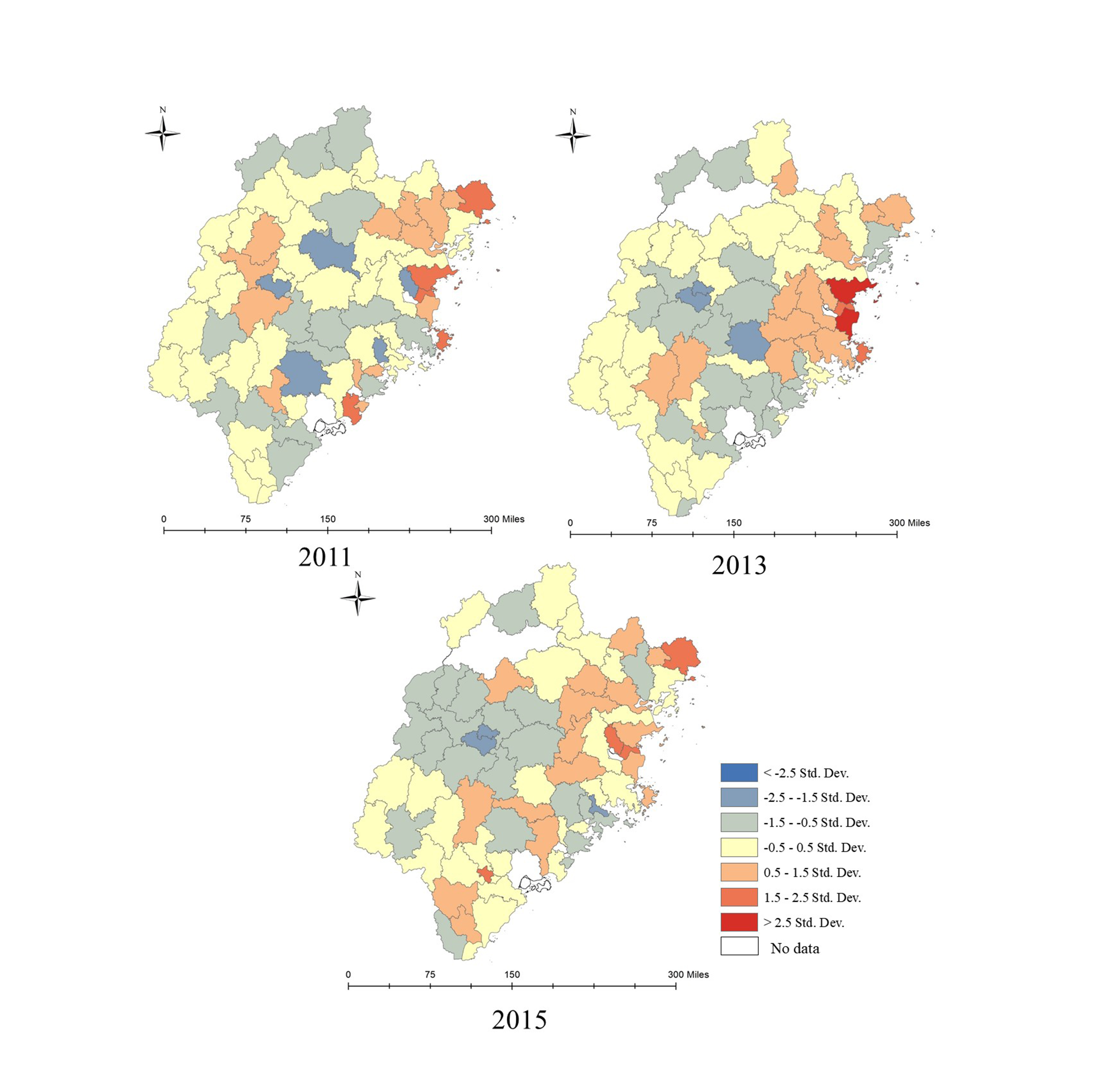

Supplement: Supplementary Figure 1 — Spatial distribution of the standard deviation of the OLS model. [file Image_1.jpg]
