# Supplementary material for: Spatial Analysis and Comparison of the Economic Burden of Common Diseases: An Investigation of 5.7 Million Rural Elderly Inpatients in Southeast China, 2010–2016
Source: Front Public Health. 2021 Nov 17;9:774342. doi: 10.3389/fpubh.2021.774342 (PMC8635627; doi:10.3389/fpubh.2021.774342)
Supplement: Supplementary Figure 2 — Spatial distribution of the coefficient of explanatory variables. [file Data_Sheet_1.PDF]

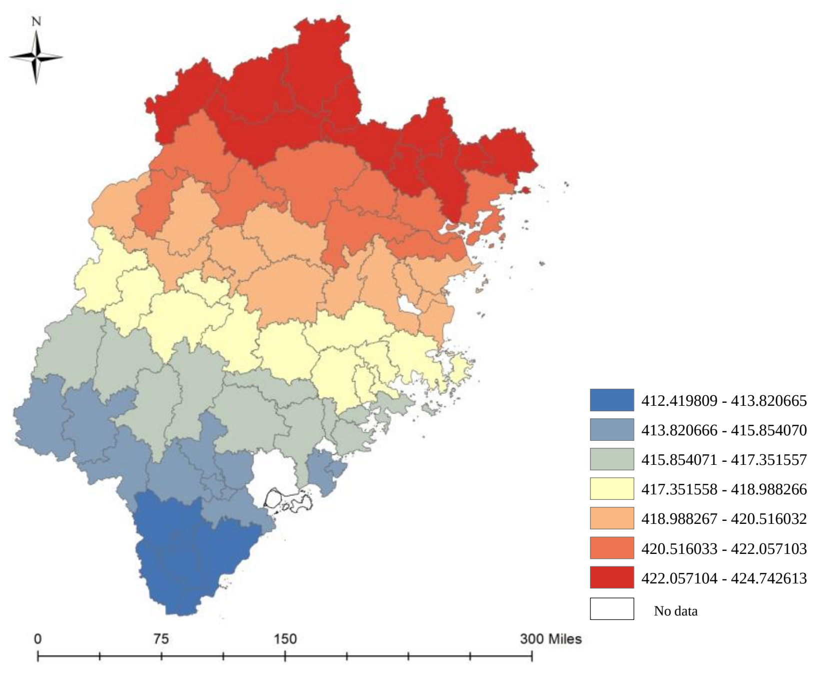

Coefficient of RD in 2011

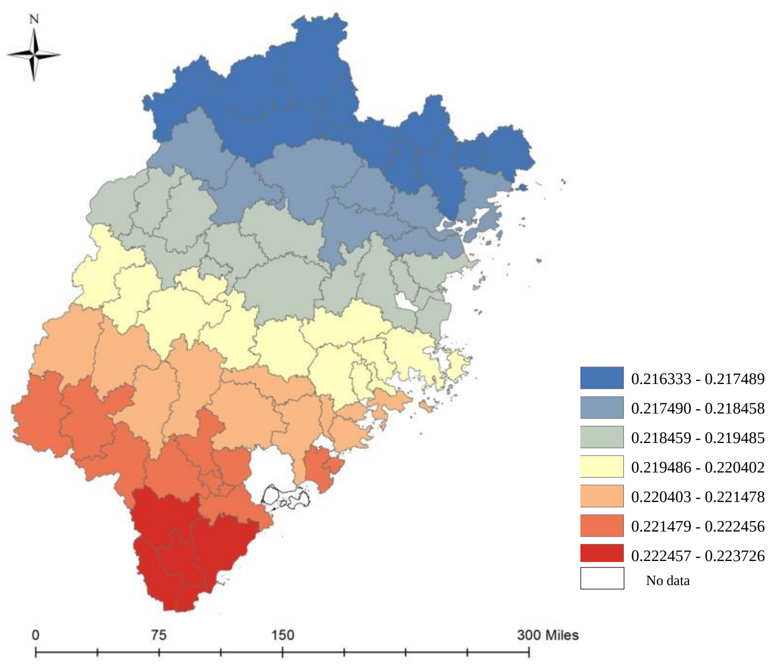

Coefficient of PCI in 2011

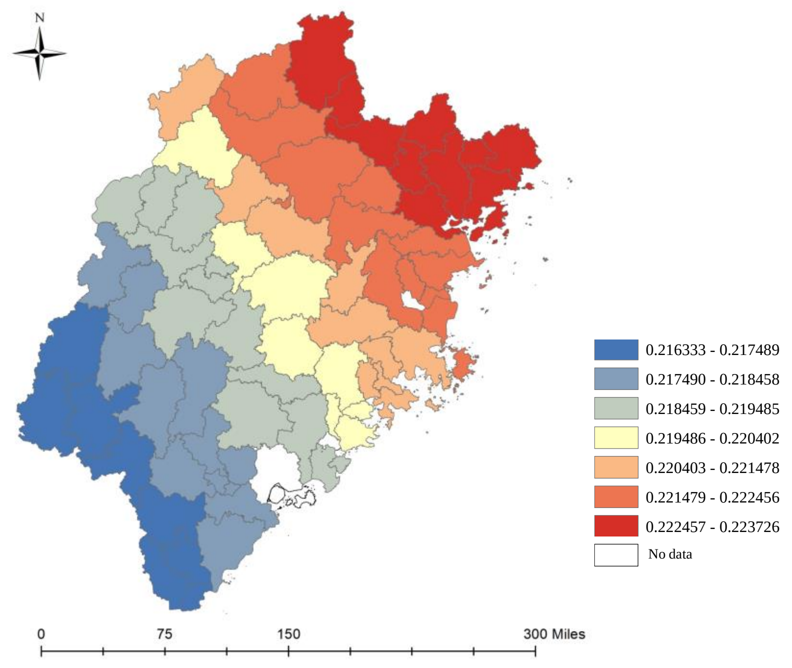

Coefficient of RP in 2011

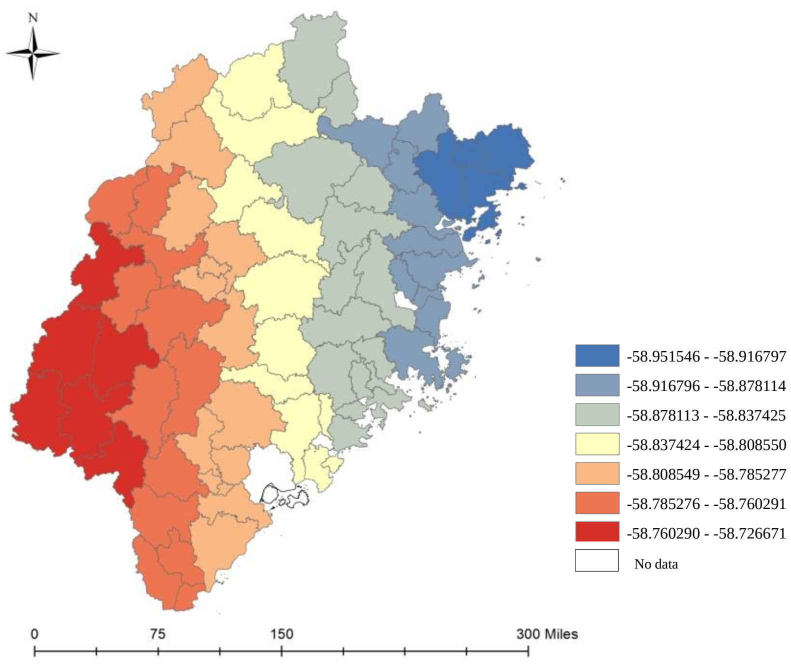

Coefficient of PTH in 2011

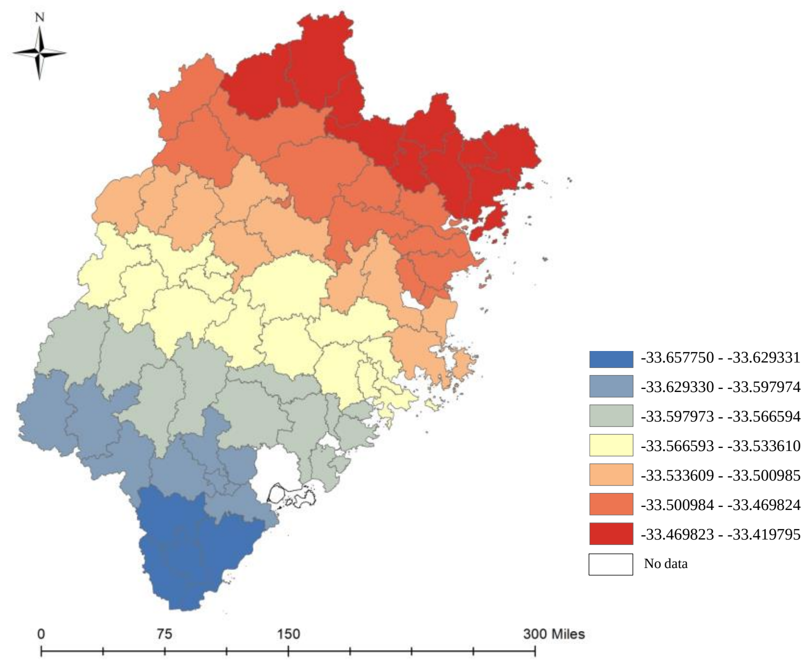

Coefficient of PCH in 2011

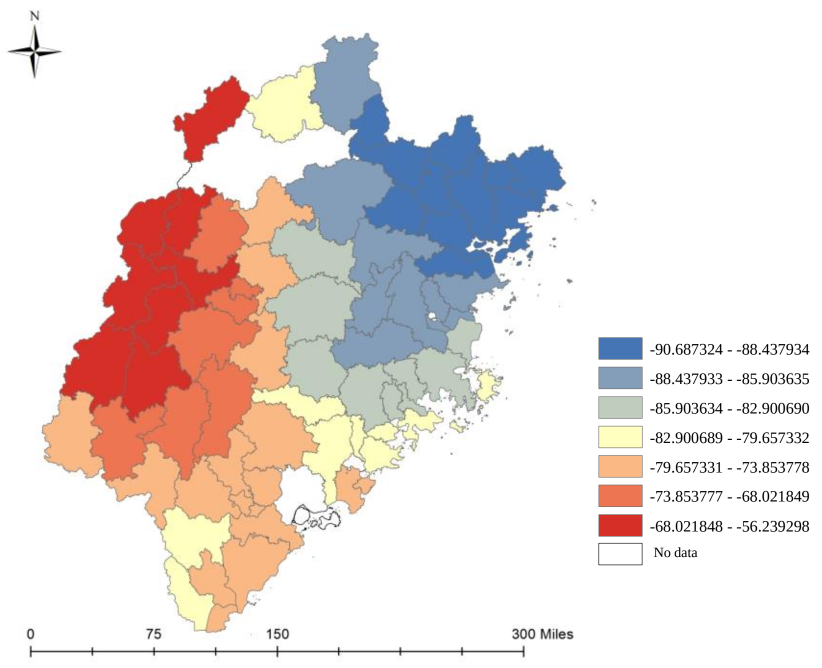

Coefficient of PTH in 2013

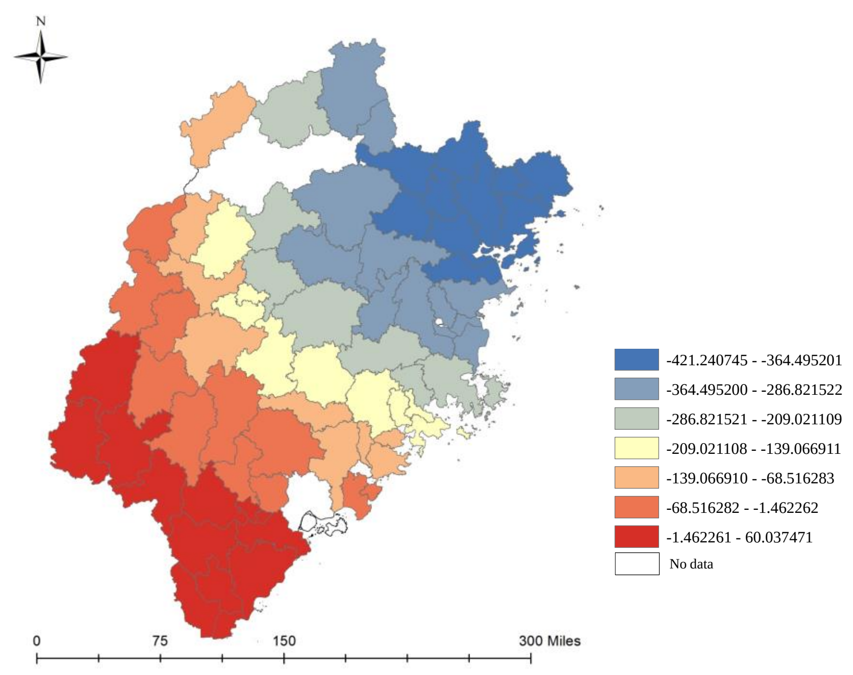

Coefficient of HB in 2013

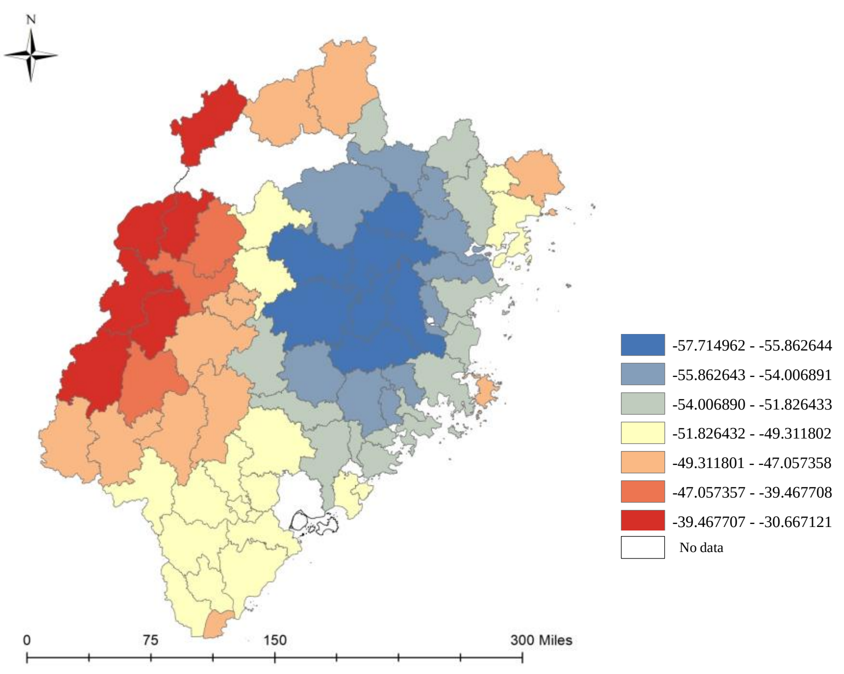

Coefficient of PCH in 2013

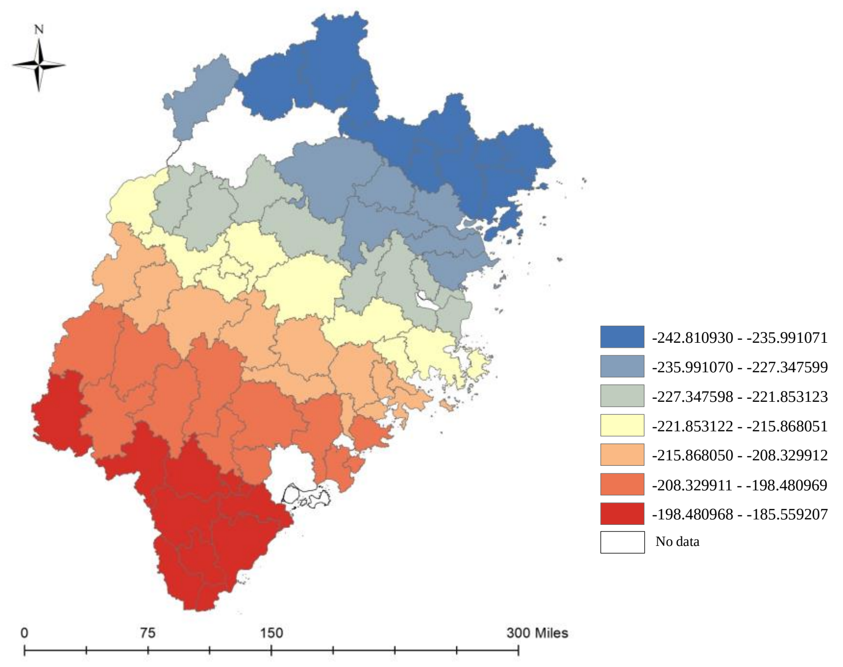

Coefficient of HB in 2015

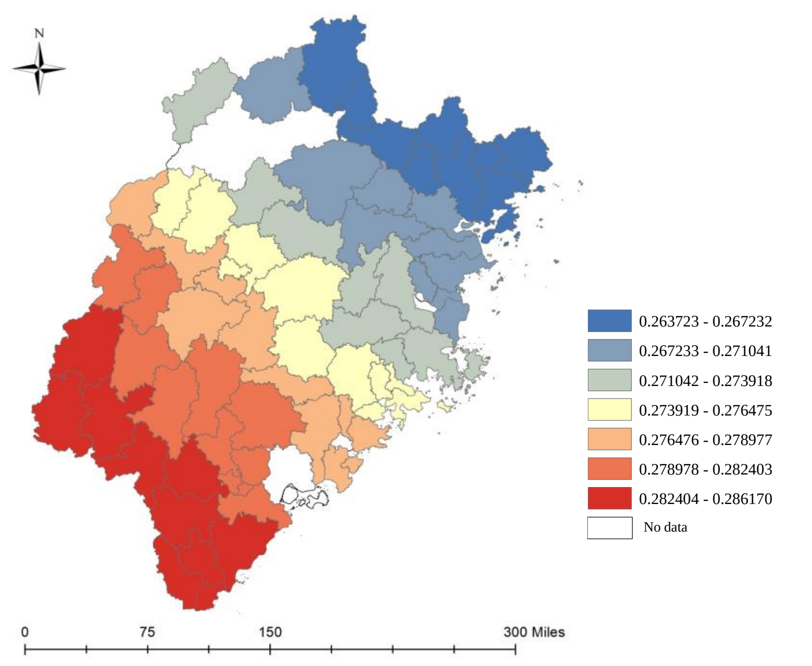

Coefficient of PCCE in 2015

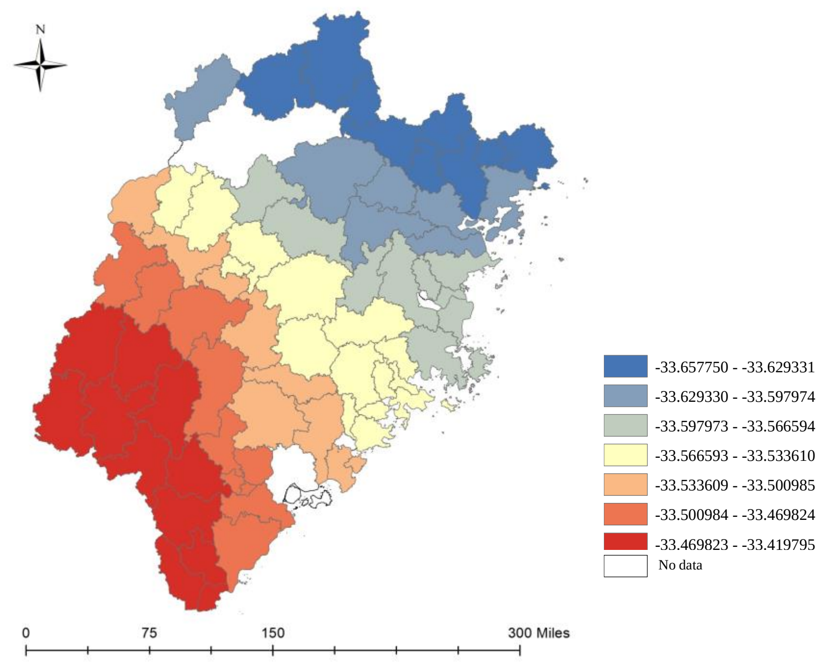

Coefficient of PCH in 2015

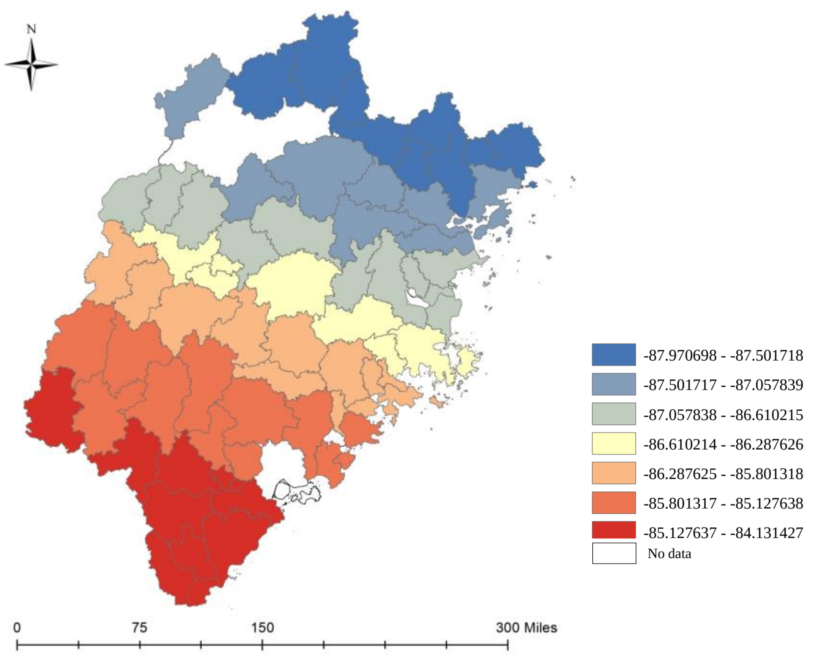

Coefficient of PTH in 2015
