# Supplementary material for: Spatial Analysis and Comparison of the Economic Burden of Common Diseases: An Investigation of 5.7 Million Rural Elderly Inpatients in Southeast China, 2010–2016
Source: Front Public Health. 2021 Nov 17;9:774342. doi: 10.3389/fpubh.2021.774342 (PMC8635627; doi:10.3389/fpubh.2021.774342)
Supplement: Supplementary Table 1 — Definition and measurements of variables. [file Table_1.docx]

**Supplementary Table 1** Definition and Measurements of Variables

| **Variable** | **Unit** | **Measurements** | **Sources** |
| --- | --- | --- | --- |
| **Dependent** |  |  |  |
| OOP | Yuan | The average value of the total hospitalization fee minus the medical insurance compensation of the elderly inpatients in each county | NRCMS |
| **Explanatory** |  |  |  |
| RP | Ten thousand population | Rural population in each county | Statistics Yearbook |
| PCI | Yuan | Per capital income of rural population in each county | Statistics Yearbook |
| PCCE | Yuan | Per capital consumption expense of rural population in each county | Statistics Yearbook |
| PGDP | Ten thousand yuan per capita | Per capital GDP of rural population in each county | Statistics Yearbook |
| RD | Kilometer per square kilometer | The total length of each county’s highway divided by its area | Statistics Yearbook |
| HB | Per thousand population | The number of hospital beds divided by total population in each county | Statistics Yearbook |
| HT | Per thousand population | The number of health technicians divided by total population in each county | Statistics Yearbook |
| PTH | Percentage | The proportion of patients hospitalized in town-level hospitals by all inpatients in each county | NRCMS |
| PCH | Percentage | The proportion of patients hospitalized in county-level hospitals by all inpatients in each county. | NRCMS |

Abbreviations: OOP, out-of-pocket; NRCMS, New Rural Cooperative Medical Scheme; RP, rural population; PCI, per capital income; PCCE, per capital consumption expense; PGDP, per capital GDP; RD, road density; HB, hospital beds; HT, health technicians; PTH, proportion of town-level hospital inpatients; PCH, proportion of county -level hospital inpatients.
